# Using a LIDAR Vegetation Model to Predict UHF SAR Attenuation in Coniferous Forests

**DOI:** 10.3390/s90301559

**Published:** 2009-03-06

**Authors:** Alan Swanson, Shengli Huang, Robert Crabtree

**Affiliations:** 1 Yellowstone Ecological Research Center, 2048 Analysis Drive, Suite B, Bozeman, MT 59718, USA; E-Mail: crabtree@yellowstoneresearch.org (R.C.); 2 NASA Ames Research Center Mail Stop 242-4, Moffett Field, CA 94035 USA; E-Mail: huang@yellowstoneresearch.org (S.H.)

**Keywords:** SAR, Lidar, Forest, Attenuation

## Abstract

Attenuation of radar signals by vegetation can be a problem for target detection and GPS reception, and is an important parameter in models describing vegetation backscatter. Here we first present a model describing the 3D distribution of stem and foliage structure based on small footprint scanning LIDAR data. Secondly we present a model that uses ray-tracing methodology to record detailed interactions between simulated radar beams and vegetation components. These interactions are combined over the SAR aperture and used to predict two-way attenuation of the SAR signal. Accuracy of the model is demonstrated using UHF SAR observations of large trihedral corner reflectors in coniferous forest stands. Our study showed that the model explains between 66% and 81% of the variability in observed attenuation.

## 1. Introduction

Synthetic Aperture Radar (SAR) signals interact with objects on the scale of their wavelength. In a forested setting, Ultra High Frequency (UHF) SAR signals from the FOliage PENetration (FOPEN) sensor (λ = 88cm) interact primarily with tree trunks, the ground and larger branches. As the signal passes through the vegetation, these interactions attenuate the signal, so that the return from objects within the canopy is diminished. Predicting this attenuation is of interest to those who wish to relate backscatter to forest parameters, users of global positioning system (GPS) equipment, and to those whose goal is the detection of targets concealed by forest.

As numerous authors have noted, UHF sensors respond strongly to forest structure [1–3]. Several models have been developed which describe in detail the complex radiometric interactions between SAR signals and a simplified model of 3D vegetation structure, which can be inverted to relate observed backscatter to forest structure attributes [4–6]. These models typically include an extinction coefficient, to be estimated indirectly, that describes the attenuation of the SAR signal as it passes through the forest canopy. Here we present a method that estimates this coefficient directly.

Numerous researchers have characterized vegetation attenuation at UHF frequencies [7–9], but they reported only the distribution of attenuation measurements and did not attempt to relate attenuation to a particular configuration of trees. One author has reported attenuation coefficient estimates for individual trees for both UHF and L-band SAR [10] but the methodology does not discriminate between stem and foliage, and has not been extended beyond individual trees.

Attenuation is a critical issue for GPS systems operating on L-band (λ = 23 cm) signals from satellites, causing poor reception in forested areas. The model we present is capable of predicting GPS signal attenuation.

For detection of objects within the forest canopy, vegetation interactions clutter the SAR image and attenuate returns from the object in question, making detection difficult. This makes prediction of target attenuation a concern of those developing and evaluating target detection algorithms.

In this paper we present a method for accurately predicting FOPEN UHF attenuation by coniferous forest canopy using a small-footprint scanning Light Detection and Ranging (LIDAR) imagery. Since the different components of vegetation attenuate UHF SAR at different rates, our method includes a forest vegetation model with two distinct components: stem and foliage. A ray tracing algorithm is used to track the path of SAR beams as they pass through the vegetation model, bounce through a corner reflector, and return to the sensor. Vegetation interactions are recorded and used to predict attenuation.

## 2. Data

### 2.1. FOPEN Data

The FOPEN sensor is a low frequency SAR sensor operating at Ultra High Frequency (UHF) (∼340 MHz) and Very High Frequency (VHF) (∼39 MHz) bands. We have focused our attenuation modeling efforts on the UHF band. The sensor is mounted on an Army RC-12 twin-engine aircraft. It is fully polarimetric at UHF frequencies. High bandwidth (∼210 MHz) and wide synthetic aperture (∼39 degrees) allow a resolution of ∼0.5 m. The wide aperture means that a given point on the ground is imaged repeatedly along the flight path of the sensor, and these lower-resolution images are combined to synthesize the final, higher resolution image.

In the summer of 2003 the FOPEN sensor was flown for 36 passes over our study area, near the northeast corner of Yellowstone National Park (YNP). The look direction of passes varied by day. One image was very blurry and was excluded from this analysis. Depression angle (angle below the horizontal) at image center ranged from 15.0 to 27.1 degrees.

**Figure 1.**
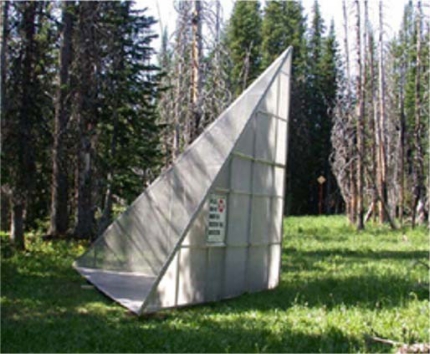
Large trihedral corner reflector. These reflectors are 5 m tall.

Twelve large trihedral radar reflectors were placed in varying degrees of vegetation obscurity during these flights. The reflectors were constructed out of aluminum mesh panels supported by a frame of tubular aluminum, and had a dimension of 5 meters. Each reflector was oriented so that its bottom plate was level and its front edge was parallel to the flight path of the sensor on that particular day. Figure 1 shows a picture of a large reflector. Of the 12 large (5 m) reflectors in place during the FOPEN flights, three were placed in open fields unobscured by vegetation and therefore considered to be control reflectors. The other ten were placed in varying degrees of vegetation obscurity. Each reflector was imaged 17 times from the west, 4–5 times from the south, and 4–5 times from the north, yielding 79 control reflector observations and 233 observations of obscured reflectors. The viewing geometry for individual reflectors was calculated from planned flight path information.

### 2.2. Field Data

At six of the reflector sites and five other sites, extensive vegetation measurements were made. At the six reflector sites a 50 m × 50 m grid was established and divided into 25 10 m × 10 m subplots. At the five other sites, a 30 m × 30 m grid was established and divided into nine 10 m × 10 m subplots. This resulted in 138 subplots containing large trees. The dominant tree species found throughout the study plots was subalpine fir (*Abies lasiocarpa*). The corners of these grids were located using differential GPS and subplot boundaries were interpolated from these locations. Within each subplot, all trees were measured. For all live and standing dead trees over 3.1 m tall, diameter at breast height (DBH), height, and species were recorded.

### 2.3. LIDAR Data

LIDAR data were collected over the study site with an Optech Airborne Laser Topographic Mapping (ALTM) 1233 sensor on August 1, 2003. The ALTM 1233 sensor is a small-footprint scanning LIDAR that utilizes a 1,064 nm wavelength yttrium aluminum garnet (YAG) laser pulsed at a repetition rate of 33 KHz. The scan angle was +/− 8 degrees and the scan frequency was 39 Hz, creating an average swath width of 422 meters. Average hit density was 0.88 hits per square meter. These data were supplied as a 32-bit floating point first return Digital Surface Model (DSM) raster at a spatial resolution of 1m. A local minima routine [11] was used to separate ground from vegetation, resulting in a Digital Elevation Model (DEM) of the ground surface and Canopy Height Model (CHM) at 1 m resolution.

## 3. Methods

Our method consists of the following steps:

- Attenuation is estimated for each obscured reflector (3.1).
- A 3D representation of foliage and stem volume is created from a small-footprint scanning LIDAR image (3.2).
- A 3D model of a 5 m trihedral corner reflector is generated and placed within the vegetation model (3.3).
- Numerous (n=1024) simulated SAR beams, evenly distributed over the opening of the reflector, are tracked as they pass through the vegetation model, interact with the corner reflector, and return through the vegetation. All vegetation interactions are recorded. This step is repeated for numerous positions along the flight path of the sensor, giving full representation to the FOPEN aperture. These values are averaged across the reflector surface and the FOPEN aperture to give mean foliage and stem obstruction for each attenuation observation (3.4).
- Mean foliage and stem obstruction are used in a linear model to predict attenuation (3.5).

### 3.1. Estimating Attenuation

Attenuation of obscured reflectors was estimated by comparing their expected brightness (in the absence of vegetation) to their observed brightness. The expected brightness was derived by modeling the observed brightness of control reflectors as a function of depression angle.

#### 3.1.1. Measuring Reflector Brightness

All reflectors' locations were visually identified in slant range single look complex (SLC) images. This is reliable because in all cases the reflectors were slightly brighter than the surrounding vegetation. Small image chips (150 by 150 pixels) centered on each reflector were extracted and upsampled 3X before measuring the peak brightness. Upsampling was accomplished by converting the image chips to frequency space using a fast Fourier transform, padding with zeroes to create a chip with a dimension of 450 by 450 pixels, then back-transforming into Euclidian geometry using an inverse fast Fourier transform. The upsampled chips were then converted to decibels, and peak brightness from a small region surrounding the reflector location was recorded.

#### 3.1.2. Estimating Attenuation

**Figure 2.**
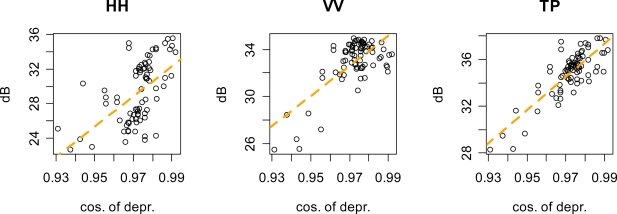
Control reflector brightness as a function of local depression angle. The x-axis is cosine of local depression angle minus 35.26 degrees, which is the fractional change in the size of the reflector opening relative to its maximum at a local depression angle of 35.26 degrees. The orange lines represent linear fits. These models, respectively, explained 31, 50 and 67 percent of the variability in control reflector brightness. Residual standard error was 2.79, 1.45, and 1.12 dB respectively.

Attenuation of obscured reflectors was estimated as the difference between expected (in the absence of vegetation) and observed brightness. Expected brightness was determined by modeling the brightness of control reflectors as a function of local depression angle, which varied between images and between reflectors within images. Since the reflectors were always oriented with their bottom plate level, changes in local depression angle affected their effective radar cross section (RCS). This effect was modeled as a linear function of *cos(depression-35.26°)*, since this is proportional to change in the size of the reflector opening as local depression angle departs from its maximum at a depression angle of 35.26°. Figure 2 shows the fit of this model for the two polarizations and for total power.

### 3.2. LIDAR Vegetation Model

Tree trunks, or stems, attenuate long wavelength SAR far more strongly than other components of vegetation [1–3]. Due to the tapered profile of stems, stem volume follows a vertically skewed distribution within the vegetation volume. Therefore we created two competing models which partition a LIDAR vegetation height image into stem and foliage components.

These models were created from the 2D LIDAR using the following scheme:

1. A 3D byte array for the area surrounding a reflector is generated to hold the vegetation model.
2. Each column of the 3D array is populated based on the corresponding height value of the 2D DSM and DEM images. Voxels (volume pixels) below the DEM are encoded as ground. Voxels between the DEM and the DSM are encoded as vegetation using an encoding scheme that identifies each voxel’s vertical position within its vegetation column. Voxels above the DSM are encoded as air.

We used two methods to model the distribution of stems within the volume of vegetation. The discrete stem model places stems discretely within the vegetation layer while the probabilistic stem model distributes stems throughout the vegetation layer. Both models will serve as input to the attenuation model we present later. Their ability to explain attenuation will provide an objective means of comparison.

#### 3.2.1. Discrete Stem Model

**Figure 3.**
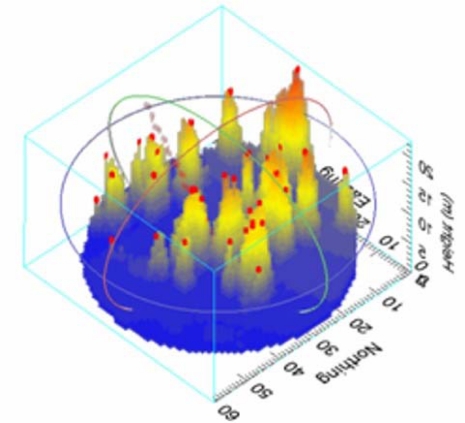
Voxel representation a forest scene with treetops identified using the local maxima filter colored red.

In high resolution scanning LIDAR imagery, individual trees are often clearly visible, especially when they stand apart from adjacent trees. The discrete stem model uses a local maxima filter to identify pixels of the CHM which are higher than all of their immediate neighbors. The vegetation columns below these local maxima are then encoded as stem. Figure 3 shows a voxel representation of a forest scene with treetops identified.

The local maxima filter can identify the location and height of stems, but tells us nothing about their shape. The stem of a tree is widest at the bottom and tapers to a point at the top. The shape of this taper has been estimated for the tree species (as a function of DBH and height) in our study area by Flewelling and Raynes [12]. We have developed a simplification of these models based on height only.

The Flewelling profile models are region- and species-specific, and require height and DBH to produce an estimated stem profile. Since height and DBH are highly correlated, we used a regression analysis of field-measured trees to model DBH as a function of height. Flewelling stem profiles for a series of stems with DBH predicted from height were created for Subalpine Fir, the most common species in our study area.

**Figure 4.**
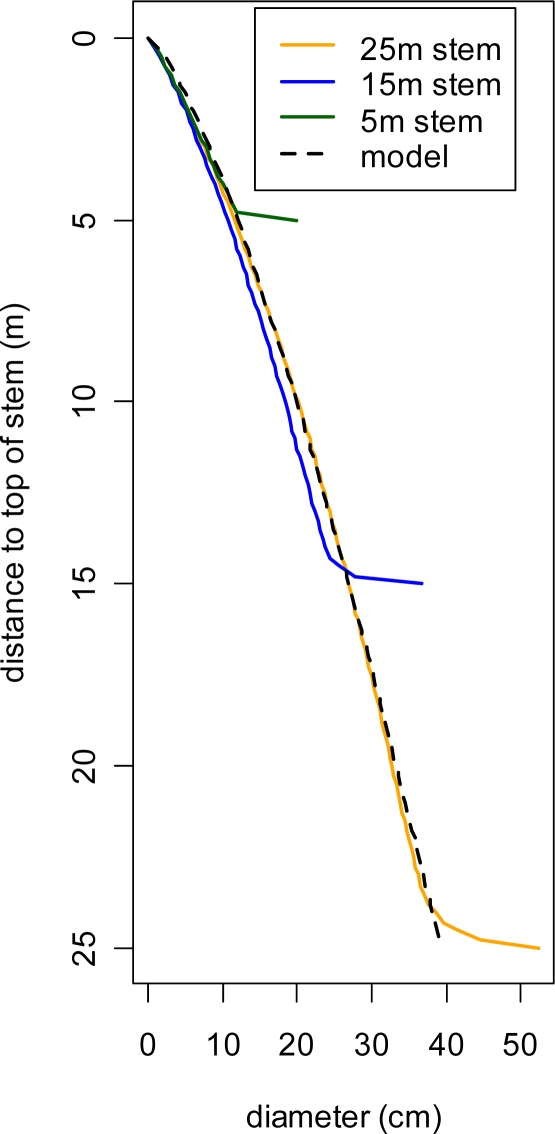
Estimated stem profiles for Subalpine firs of five different heights. These were generated using Flewelling profile models for trees with DBH proportional to height.

Examination of these profiles, shown in Figure 4, suggested that a simple model of diameter as a parabolic function of distance to the tree top could be used to approximate the Flewelling stem profiles. The corresponding equation is: *dia* = *a*·*x^b^*. The method of least squares was used to fit this model to the profile model of a 25 m stem, resulting in parameter estimates of *a_1_*=*0.03649* and *b_1_*=*0.738*. We used this parabolic approximation to estimate stem diameter for each stem voxel in the 3D vegetation model. Stems were modeled as centered within their voxel column and the remaining voxel volume (stems did not exceed 1 m in diameter) was considered to be foliage.

#### 3.2.2. Probabilistic Stem Model

Comparison to field data revealed that the local maxima filter was missing a large number of trees. Individual trees distinct from their neighbors are easily identified, but trees very close to a taller neighbor were not reliably separated. To overcome this difficulty we developed a ‘probabilistic’ stem model that distributes stem biomass throughout the vegetation volume.

This model is similar to the discrete stem model, except that instead of discretely placing stems under local maxima, every column of vegetation is modeled as containing a stem. As with the discrete stem model, a parabolic model is used to describe stem diameter, but with coefficients that were derived from field data using a two step procedure as follows.

For each of our 138 10 m × 10 m forest plots, Flewelling stem profile models [12] were used to estimate total stem volume. The LIDAR CHM was then extracted for each plot and the parabolic approximation was applied to derive estimates of stem profile for each vegetation column, which were then integrated to estimate stem volume. An optimization routine was used to find the parabolic exponent which maximized the correlation between the field and LIDAR estimates of stem volume. A value of *b*=*0.77* maximized this correlation, which is very close to the value used for the discrete stem model. The coefficient *a* was then estimated by regressing field stem volume on the LIDAR estimates with the exponent fixed at 0.77. This resulted in a coefficient estimate of *a* = *0.14*.

### 3.3. Reflector Model

**Figure 5.**
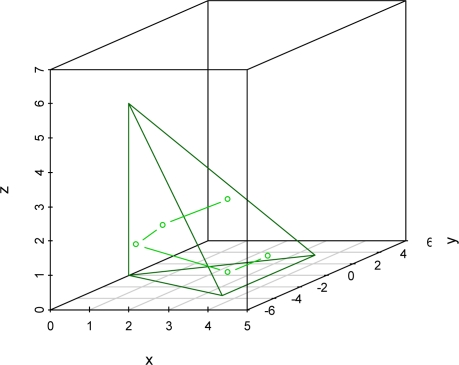
3D diagram showing the path of a simulated sar beam as it passes through a modeled trihedral reflector.

To simulate the shape and reflective properties of the trihedral reflectors, we developed a 3D model. This model specifies the three active planes of the reflector and distributes 1,024 nodes over the opening plane using a recursive splitting algorithm. A ray tracing algorithm was developed to track the reflection of simulated SAR beams off the active planes. Figure 5 shows the path of a simulated SAR beam as it passes though a modeled reflector. The ray tracing algorithm allowed us to identify active nodes on the reflector opening for any sensor geometry, and to identify the exit point of a SAR beam entering at any active node.

### 3.4. The Attenuation Model

#### 3.4.1. Overview

To model attenuation of the SAR signal, we simulate the passage of SAR beams though the LIDAR vegetation models to estimate a series of stem and foliage path length over the SAR aperture. This can be interpreted as the average (averaged over the reflector opening) distance of stem and foliage that a beam must pass through on its way to and from the reflector. Since available power varies throughout the aperture, set weights, which mimic the pattern of variations in power, were derived. Weighted mean stem and foliage path lengths are then used a linear model to predict attenuation.

#### 3.4.2. Recording of Vegetation-SAR interactions

Synthetic aperture radar integrates information from a series of points along a flight path to form an image. With the FOPEN sensor, the integration angle is approximately 39 degrees, so vegetation in a wedge-shaped area in front of the reflector can potentially contribute to attenuation. To simulate the motion of the sensor, we made vegetation measurements for 80 points along the aircraft flight line, representing the full aperture. At each point along the flight line we tracked 1,024 simulated SAR beams distributed over the opening plane of the 3D reflector model. For each beam that successfully passed through the reflector model, all vegetation interactions were recorded along its path to the reflector and its return to the sensor. To perform this calculation, each foliage voxel that a beam passed through was examined, and the exact distance of both foliage and bole were calculated based on the bole diameter in that voxel (assumed to be in located the center of the voxel) and the trajectory of the beam. Meters of foliage and meters of stem were estimated for each valid beam, and these values were averaged over all valid beams at each azimuth increment. This resulted in a matrix containing 80 measurements of stem and 80 measurements of foliage obstruction for each reflector observation.

#### 3.4.3. Antenna pattern Estimation

Available power of a SAR sensor varies throughout the aperture, with the most power available in the center. This variation in power is due to a number of factors:

- The antenna puts out greater power mid-aperture than at the edges.
- A target is further from the sensor at the edges of aperture and thus receives less energy.
- Taylor weighting, applied during image formation, down-weights the edges of aperture.
- The RCS of a trihedral reflector is largest mid-aperture, tapering near the edges.

Since more power is available near the center of the aperture, vegetation in that region has a stronger effect on attenuation. To account for this, our model weights vegetation interactions based on their position in the aperture. We have taken an empirical approach to derive weights based on available power, using a series of directional filters to estimate subaperture response of control reflectors as a function of azimuth.

**Figure 6.**
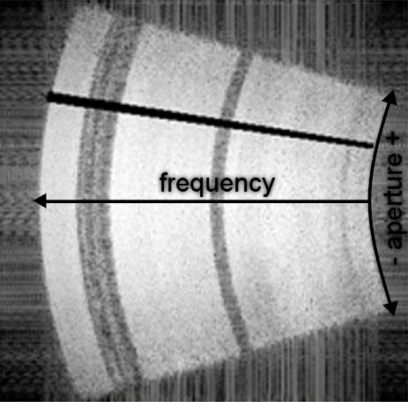
Frequency space image of an unobscured trihedral reflector. The frequency extends from 235–445 Mhz and aperture extends from −19.75 to +19.75 degrees. The black region is 1-degree slice centered on 10.25 degrees.

**Figure 7.**
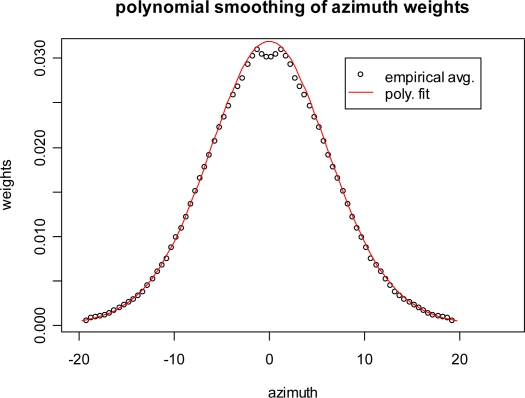
Subaperture weights. The points are the empirical average of subaperture observations on eighteen control reflectors. The line represents a 6th degree polynomial fit of the points.

Our method of estimating subaperture response is similar to that used by Runkel *et al*. [13]. Image chips surrounding a reflector are extracted and transformed into frequency space using a Fast Fourier Transform (FFT). In frequency space, the image chip takes on polar coordinates with angle corresponding to the azimuth and radius corresponding to frequency. The origin and angular scale in frequency space are estimated and all but the desired aperture slice is masked out. The chip is upsampled by padding with zeroes and is transformed back into Euclidian space using a reverse FFT. Peak brightness is then measured in the power scale. Figure 6 shows an example of a frequency space image with a 1 degree slice masked out. This figure shows how 1 degree slices are mapped, but for estimation of antenna pattern we mask out all but the 1-degree slice. We performed this processing method for non-overlapping 1 degree increments from a sample (n = 18) of control reflector observations. To generate a smooth and symmetrical curve, these 18 observations were averaged as a function of distance to aperture center. The resulting curve still contained, so it was fit with a 6th degree polynomial. Figure 7 shows the average values and the polynomial fit. The fits were standardized so that they summed to one and used as weights to calculate weighted mean foliage and stem interactions, the predictors for the attenuation model.

### 3.5. Estimating Attenuation Coefficients

Mean foliage and stem were used in a linear model to predict attenuation. Errors for repeated observations of the same reflector (from a given look direction), although varying in depression angle, can be assumed to lack independence, since geolocational errors and errors in the LIDAR vegetation models will affect individual repeated observations similarly. This lack of independence violates the assumptions of simple linear regression, but can be modeled efficiently by including a random effect for each reflector/look direction combination. This model is specified as:

$$y_{\mathrm{ij}}=\beta_{0}+\beta_{1}\cdot {\mathrm{foliage}}_{\mathrm{ij}}+\beta_{2}\cdot {\mathrm{stem}}_{\mathrm{ij}}+b_{i}+\varepsilon_{\mathrm{ij}},\;\;\;\;b_{i}∼N(0,\sigma_{b}^{2}),\;\;\;\;\varepsilon_{\mathrm{ij}}∼N(0,\sigma^{2})$$

where *i* indexes the reflector/look combination and j indexes observations within that combination. In this model, the term, *b_i_*, fits a different mean value for each reflector/look combination. Rather than using a fixed effect for each combination, in the mixed effects model we fit this effect as zero-centered Gaussian random variable with only one parameter: *σ_b_*, its standard deviation. Coefficient estimates (and predictions) from such a mixed effects model will be similar to those from a simple linear regression model, but with standard errors that better reflect the correlated error structure of the data.

This mixed linear model was fit with the R language for statistical computing [14] using the nlme library [15]. Models were fit for the two polarizations and for total power, and for both vegetation models. A third set of models were fit to total vegetation path length, the sum of stem and foliage path lengths. The method of restricted maximum likelihood (REML) method was used for all model fits, except that comparisons between models were performed using maximum likelihood (ML) fits. REML produces unbiased estimates of variance parameters, but ML methods allow for statistically valid comparisons of models with different predictors [15]. Model comparisons were made using Akaike’s Information Criterion (AIC) [16], which is calculated as:

$$\mathrm{AIC}=-2\cdot \mathrm{LogLik}+2\cdot n_{\mathrm{par}}$$

where LogLik is the log likelihood and n_par_ is the number of parameters in the model. A lower AIC indicates a better model fit.

## 4. Results

**Figure 8.**
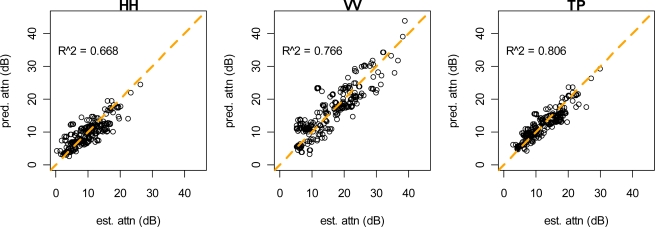
Attenuation model predictions using the discrete stem model.

**Figure 9.**
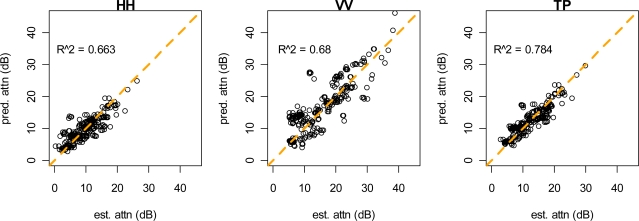
Attenuation model predictions using the probabilistic stem model.

Vegetation attenuation coefficients were estimated for the HH polarization, the VV polarization, and for total power (Table 1). These coefficients were estimated for both stem models and for the vegetation model with no stem component. Coefficient estimates are reported in Table 1 and plots of predicted vs. observed attenuation are shown in Figures 8 and 9. The highest overall attenuation and the largest stem attenuation coefficients were found for the VV polarization. These models explained between 66% and 81% of the variation in observed attenuation. Residual standard error was between 1.19 and 1.52 dB, which represents attenuation measurement error. This compares well with residual standard error from our model of control reflector brightness (1.12–2.79 dB). Standard error of the random effect for each reflector/look combination ranged from 2.13–5.15 dB, which represents variation due to geolocation error, inaccuracy of LIDAR data, and variation not explained by the model. The R-squared values in Table 1 were derived from making population-level predictions (the random effect was set to zero) and calculating the squared correlation coefficient between these predications and estimated attenuation.

## 5. Discussion and Conclusions

In this paper we present a model that, given a 3D canopy height model, can be used to predict the attenuation of a SAR signal as it passes through a forest canopy. Our attenuation coefficient estimates are roughly similar to those reported by Cadvar [10] who, using vertically polarized UHF, estimated the attenuation coefficient for pine (no species given) to be 1.8 dB/m. Our estimate, from the model with no stem component, was 0.76 dB/m. This discrepancy could be due to random error, a difference in tree species, or a difference in the stem to foliage ratio of the trees measured.

Our results show greater attenuation in vertical polarization than for horizontal. This is in agreement with the literature, where it has been attributed to the greater cross-sectional area of tree-trunks in the vertical plane [7,8]. In our modeling results, the stem attenuation coefficient is consistently higher for the vertical polarization than for the horizontal. In fact, the stem coefficient is not statistically significant (α = 0.05) for the HH polarization but highly significant for VV.

For comparison of stem models we focus on the VV polarization since stem is not statistically significant in the HH models. The AIC values in Table 2 indicate that the probabilistic stem model better fits the data than the discrete stem model for the VV polarization. The stem attenuation coefficients for the probabilistic stem model are much more realistic since we know that the discrete model significantly underestimates forest stem volume. Interestingly, the discrete stem model result in better R-squared values despite inferior AIC values. This is because the likelihood function (on which the AIC score is based) for mixed effects models places a greater penalty on residual standard error (RSE) than on the standard error of the random effect. The discrete model has a lower SE for the random effect but a higher RSE, so it produces better population-level predictions (which do not include random effects for individual reflector/look combinations), but has a higher (worse) AIC score.

Our coefficient estimates suffer somewhat from multicollinearity between the stem and foliage path-length estimates. The correlation coefficients for these two metrics are 0.92 for the probabilistic stem model and 0.81 for the discrete stem model. This results in instability of our coefficient estimates, but cannot be avoided given the design of our study.

In conclusion, our model does a very good job of predicting UHF attenuation. We prefer the discrete stem model for making attenuation predictions with our LIDAR data, but place more trust in the stem attenuation coefficient estimates from the probabilistic model. For making predictions with LIDAR data of differing resolution, we would prefer the probabilistic model for prediction since the discrete stem model would place a greater or lesser number of stems.

## Figures and Tables

**Table 1.** This table gives coefficient estimates for a series of linear mixed-effects model that were used to predict attenuation as a function of foliage and stem obscuration. For each polarization and total power, three stem models were compared. R-squared is given for predictions which do not include random effects.

**Polarization**	**Stem model**	**Foliage coefficient**	**Foliage p-value**	**Stem coefficient**	**Stem p-value**	**AIC**	**R-squared**
HH	prob	0.264	0.0000	32.71	0.039825	919.0	0.663
HH	discrete	0.369	0.0000	11.87	0.593	922.8	0.668
HH	no stem	0.392	0.0000	NA	NA	921.1	0.660
VV	prob	0.293	0.0000	119.36	0.0000	959.6	0.680
VV	discrete	0.600	0.0000	81.38	0.0040	980.5	0.766
VV	no stem	0.761	0.0000	NA	NA	986.0	0.773
TP	prob	0.286	0.0000	41.87	0.0017	839.7	0.784
TP	discrete	0.414	0.0000	18.66	0.3021	847.6	0.806
TP	no stem	0.450	0.0000	NA	NA	846.6	0.794
